# A Manual of Bacteriology

**Published:** 1899-12

**Authors:** 


**A Manual of Bacteriology.** By Richard T. Hewlett, M.D. Pp. viii., 439. London: J. & A. Churchill. 1898.

There are several novel features in this book which will commend it to the student of bacteriology and to the clinician. The early chapters are devoted to the description of laboratory work, preparation of media, and the methods of cultivating and staining bacteria. There then follow detailed descriptions of the several varieties of pathogenic organisms. At the end of each chapter there is added a series of paragraphs on clinical diagnosis in which very full details are given of the methods to be pursued in taking cultures from the patient, and of the best ways of preparing and examining these cultures. The directions given are clear and concise, and we regard this new departure as one of great practical value.

It is satisfactory, too, to find space devoted to the bacteriology of such diseases as variola, conjunctivitis, diarrhœa, noma, otitis media, ozœna, rheumatoid arthritis, and syphilis. Chapters are also given to the hyphomycetes, the blastomycetes, and the protozoa. Under the first named group are described the ringworm and thrush parasites, together with minute directions for the preparation of cultures and microscopic specimens of these organisms. Mention is made of the pathogenic nature of certain of the blastomycetes and of their relationship to new growths. Recent investigations of Sanfelice in Italy, and of Russell and Plimmer in this country, would point to the cancer organism being included in this group; but we note that malignant disease is dealt with under the heading of diseases of uncertain etiology.

The information in the appendix relating to the nature and preparation of antitoxins and vaccines is full and abreast of present knowledge. The book is excellent throughout; the only alteration which we could have wished for is a fuller description of the microscopic appearances of the various organisms. For instance, the Klebs-Löffler bacillus is described as "a small delicate bacillus with rounded ends," a description which is not calculated to facilitate the recognition of many or indeed the majority of forms met with.

